# Prevalence of cardiovascular risk factors and the metabolic syndrome in middle-aged men and women in Gothenburg, Sweden

**DOI:** 10.1186/1471-2458-8-403

**Published:** 2008-12-08

**Authors:** Lennart Welin, Annika Adlerberth, Kenneth Caidahl, Henry Eriksson, Per-Olof Hansson, Saga Johansson, Annika Rosengren, Kurt Svärdsudd, Catharina Welin, Lars Wilhelmsen

**Affiliations:** 1 Department of Medicine, Lidköping Hospital, Lidköping, Sweden; 2 Cardiovascular Institute, Sahlgrenska Academy, University of Gothenburg, Sweden; 3 AstraZeneca, Mölndal, Sweden; 4 Karolinska Institute, Stockholm, Sweden; 5 Department of Public Health and Caring Sciences, Uppsala University, Sweden; 6 Institute of Health and Care Sciences, Sahlgrenska Academy, University of Gothenburg, Gothenburg, Sweden

## Abstract

**Background:**

Random samples of 50-year-old men living in Gothenburg have been examined every 10^th ^year since 1963 with a focus on cardiovascular risk factors. The aims of the study were to acquire up-to-date information about risk factors in the fifth cohort of 50-year-old men and women, to re-examine those who were 50 years of age in 1993, and to analyse the prevalence of the metabolic syndrome (MetSyn) using different definitions.

**Methods:**

A random sample of men and women born in 1953 were examined in 2003–2004 for cardiovascular risk factors. Men born in 1943 and that participated in the examination in 1993 were also invited. Descriptive statistics were calculated.

**Results:**

The participation rate among men and women born in 1953 was 60 and 67% respectively. Among men born in 1943, the participation rate was 87%. The prevalence of obesity was from 15 to 17% (body mass index, BMI ≥ 30) in the three samples. The prevalence of known diabetes was 4% among the 50-year-old men and 6% among the 60-year-old men, and 2% among the women. Increased fasting plasma glucose varied substantially from 4 to 33% depending on cut-off level and gender. Mean cholesterol was 5.4 to 5.5 mmol/l. Smoking was more common among women aged 50 (26%) than among men aged 50 (22%) and 60 years (15%). The prevalence of the MetSyn varied with the definition used: from 10 to 15.8% among the women, from 16.1 to 26% among 50-year-old men, and from 19.9 to 35% among the 60-year-old men. Only 5% of the men and women had no risk factors.

**Conclusion:**

This study provides up-to-date information about the prevalence of cardiovascular risk factors and the MetSyn in middle-aged Swedish men and women. Different definitions of the MetSyn create confusion regarding which definition to use.

## Background

Since the landmark Framingham Heart study in 1948 [1], there have been several hundred prospective cohort studies on cardiovascular disease and associated risk factors.

In Gothenburg the first cohort of 50-year-old men (the Study of Men Born in 1913) was examined in 1963 [2]. Younger cohorts of 50-year-old men (i.e. men born in 1923, 1933, and 1943) have later been examined every 10^th ^year [3-5].

The present study adds new data by including the 5^th ^cohort of 50-year-old men (men born in 1953). We have also examined 50-year-old women (born in 1953) in addition to a follow-up examination of the 4^th ^cohort of 50-year-old men (born in 1943), now aged 60 years.

With an increasing prevalence of excess body weight and obesity in the population, the metabolic syndrome (MetSyn) has attracted considerable attention during the past decade as an important risk factor in cardiovascular disease. There are at least five definitions of Metsyn [6], which create considerable confusion regarding which definition to use. Furthermore, with so many definitions, it is difficult to obtain consistent research results.

The aim of the present study was twofold: (1) to acquire current information about risk factors in cardiovascular disease in a middle-aged Swedish population and (2) to analyse the prevalence of the MetSyn using three popular definitions.

## Methods

### Participants

The study population consists of three cohorts: one third of all men (n = 993) and women (n = 994) born in 1953 and living in Gothenburg in 2003 were randomly sampled from the population register and invited to the examination. Gothenburg, which is a maritime and industrial city on the West coast of Sweden, is the second largest city in Sweden with approximately 450,000 inhabitants. The third cohort, men born in 1943, was a random sample in 1993 and now consists of all persons who were examined in 1993 (n = 798, 55% of those invited), except for those individuals that had died (n = 34) or moved abroad (n = 15). This leaves 749 men, now aged 60 years that were invited to participate in the present study. Based on those individuals examined, the participation rate was 60% (595 of 993) among men born in 1953, 67% (667 of 994) among women born in 1953 and 87% (655 of 749) among men born in 1943.

### Examination procedures

The examinations took place between August 2003 and December 2004. All participants were mailed a questionnaire on smoking habits and physical activity during leisure time. Each item was rated on a scale from 1 to 4, where 1 = no physical activity, 2 = moderate activity (e.g. walking, riding a bicycle and light gardening) for a minimum of 4 hours per week, 3 = regular, strenuous activity for a minimum of 3 hours per week and 4 = athletic training (competitive sports regularly). Regular smoking was defined as smoking at least one cigarette per day. Ex-smokers were defined as having quit smoking at least one month before they mailed the questionnaire. A snuff taker (snuffer) is a person who uses snuff (wet tobacco) daily.

The participants also answered questions about chest pain, psychological stress, family history of cardiovascular disease and cancer (parents and siblings), previous and current medical history and ongoing medication. Psychological stress was rated on a six-point scale with 0 = no stress, (1–3 = various grades of intermediate stress), 4 = continuous stress during the past year and 5 = continuous stress during the past 5 years. Diabetes was defined as having a physician's diagnosis of diabetes. Hypertension was defined as a physician's diagnosis and/or systolic blood pressure ≥ 140 and/or diastolic blood pressure (phase 5) ≥ 90 (physician measurement) and/or treatment for hypertension. Individuals who returned the questionnaire were invited to the examination, which was performed in the morning after an overnight fast. One reminder was sent out to the participants who did not return the first questionnaire but after that no further action was taken. The results are based solely on those persons that were examined.

The study was done in the morning. The participants were asked to fast overnight. A question relating to when they had last eaten revealed that close to 90% had complied with the request to fast. A study nurse measured height (cm) and weight (kg) with indoor clothing and without shoes. Waist circumference was measured at the level of the umbilicus (cm) and hip circumference at the level of the anterior iliac crest (cm) with the participant standing and breathing normally. After five minutes of rest, blood pressure was measured automatically in the right arm in the seated position with the OMRON 711 monitor. A 12-lead electrocardiogram was recorded with the participant relaxed and supine. Blood samples (fasting state) were taken for analysis of plasma glucose, serum total cholesterol, high-density lipoprotein (HDL) cholesterol and serum triglycerides (standard methods at the accredited university hospital laboratory in Gothenburg). During 2002, the analysis equipment at the laboratory was upgraded from Hitachi 917 Roche to Modular Roche, which resulted in an 11% increase of the mean HDL cholesterol levels [Flenner E, personal communication]. Low-density lipoprotein (LDL) cholesterol was calculated using the Friedewald formula [7]. Blood samples were frozen (-70°C) until further analysis.

After the first part of the study was completed, the participants were served a light breakfast. During breakfast, they completed another questionnaire on social and psychosocial factors, social network [8], education, working times, various complaints, sleeping habits and self-ratings on a seven-point scale regarding their health, economy, family situation, memory, energy, sleep, ability to handle stress, and simultaneous capacity [9].

A physician administered a structured interview after breakfast. The same physician also checked the questionnaires. The physician measured blood pressure using exactly the same method as in 1963 [2], i.e. with a mercury sphygmomanometer (cuff size 12 × 23 cm) in the right arm after five minutes of rest with the participant in the seated position. If potential medical problems were identified, the participants were referred for further work-up (severe hypertension, chest pain or other alarming symptoms). All participants received a letter with the results from the examination and, if needed, advice about lifestyle changes.

The review board of the Ethics Committee at the University of Gothenburg approved the study. All participants signed a written informed consent form.

### Statistical methods

The analyses were conducted using the SAS statistical software package [10]. Descriptive statistics were used. The prevalence of the MetSyn was calculated based on three definitions recently reported in the literature (Table 1).

**Table 1:** Three definitions of the metabolic syndrome.

**NCEP 2001, ref 12**	**AHA 2005, ref 6**	**IDF 2005, ref 13**
At least 3 of the following:	At least 3 of the following:	Waist circumference (Euripides) ≥ 94 cm (men), ≥ 80 cm (women) plus any 2 of the following:
1. Fasting P-glucose ≥ 6.1 mmol/l (≥ 110 mg/dl)	1. Fasting P-glucose ≥ 5.6 mmol/l (≥ 100 mg/dl) or drug treatment for elevated glucose.	1. Fasting p-glucose ≥ 5.6 mmol/l (≥ 100 mg/dl) or known type 2 diabetes
		
2. Blood pressure ≥ 130/≥ 85	2. Systolic BP ≥ 130 or diastolic BP ≥ 85 or treatment for hypertension	2. Systolic BP ≥ 130 and/or diastolic BP ≥ 85 or treatment for hypertension
		
3. Triglycerides ≥ 1.7 mmol/l (≥ 150 mg/dl)	3. Triglycerides ≥ 1.7 mmol/l (≥ 150 mg/dl) or drug treatment for elevated triglycerides	3. Triglycerides ≥ 1.7 mmol/l (≥ 150 mg/dl) or specific treatment
		
4. HDL-cholesterol <1.03 mmol/l (<40 mg/dl, men) or <1.29 mmol/l (<50 mg/dl, women)	4. HDL-cholesterol <1.03 mmol/l (<40 mg/dl, men) or <1.29 mmol/l (<50 mg/dl, women) or drug treatment for low HDL-cholesterol	4. HDL-cholesterol <1.03 mmol/l (<40 mg/dl, men) or <1.29 mmol/l (<50 mg/dl, women) or specific treatment
		
5. Waist circumference >102 cm (men), >88 cm (women)	5. Waist circumference ≥ 102 cm (men), ≥ 88 cm (women)	

## Results

### Demographic data

Demographic data are shown in Table 2. Higher education (university/college) was most common among the 50-year-old women. A higher proportion of the 50-year-olds (men and women) were born abroad (23%) as compared with the 60-year-old men (16%).

**Table 2:** Demographic data, self reported diseases, family history, and life style habits (%).

	**Men** born in 1953	**Women** born in 1953	**Men** born in 1943
	**50 years old**	**50 years old**	**60 years old**
	n = 595	n = 667	n = 655
**Demographics**			
Married/cohabiting	71.6	62.5	76.2
Divorced	13.5	19.7	11.2
University/college	35.4	42.5	28.1
Working^a^	82.2	72.8	65.5
Retired	4.7	8.6	16.8
Self-employed	21.4	6.5	23.1
Born in Sweden	77.0	76.7	84.4
Born in Europe^b^	12.3	17.4	12.5
Born outside Europe	10.8	5.9	3.1
**Diseases and the family history**			
Myocardial infarction	1.2	0.5	6.7
Coronary by-pass surgery	0.8	0	3.4
Percutaneous coronary intervention	0.3	0.2	3.8
Atrial fibrillation^c^	1.0 (0.7)	0.2 (0.2)	4.4 (1.1)
Stroke	0	0.5	2.4
Family history of myocardial infarction^d^	35.5	38.2	35.9
Family history of stroke^d^	25.0	27.3	26.4
Family history of diabetes^d^	25.0	23.2	24.0
**Life style habits**			
Regular smokers	21.5	26.1	15.1
Ex-smokers	41.0	36.3	47.8
Snuff, daily use	18.7	2.9	16.8
Ex-snuffers	15.9	2.4	9.3
Coffee daily	89.1	93.4	96.6
Continuous stress^e^	17.3	22.8	10.3
No physical exercise, leisure time	17.7	13.8	11.6
Physical exercise, leisure time^f^	23.7	23.9	23.8

^a^Full time or part time work ^b^Outside Sweden ^c^Figures within parenthesis are the frequencies of atrial fibrillation recorded at the examination ^d^The family history among fathers or mothers or siblings as reported by the participants ^e^Continuous stress as reported by the participant during the last year or up to the last 5 years ^f^Regular or intense

### Self-reported diseases and family history

In one of the questionnaires the participants were asked about various common diseases (Table 2). Cardiovascular diseases and intervention procedures were more commonly reported by the 60-year-old men than by the 50-year-old men and women. Between 36 and 38% of the participants reported a family history of myocardial infarction, 25–27% reported a family history of stroke, and 23–25% reported a family history of diabetes.

### Anthropometric measurements

Details on anthropometric variables are given in Table 3. The prevalence of obesity (Body mass index [BMI] ≥ 30 kg/m^2^) was 15% among 50-year-old men and women and slightly higher (16.6%) among 60-year-old men. Using the WHO cut-off for waist/hip ratio (>0.85 for women, >0.90 for men, ref. 11), 38% of the women and 73% of the 50-year-old men and 79% of the 60-year-old men had abdominal obesity. Using the AHA criteria (Table 1, ref. 6, only waist circumference), 30% of the women and 22 and 30% respectively of the 50- and 60-year-old men had abdominal obesity. Using the International Diabetes Federation (IDF) criteria (Table 1, only waist circumference) the corresponding figures for women and men were 56% and 51–61% respectively.

**Table 3:** Anthropometric data (means and standard deviation) and cut-off levels for body mass index (BMI), waist circumference and waist/hip ratio.

Variable	**Men** born in 1953 **50 years old** n = 595	**Women** born in 1953 **50 years old** n = 667	**Men** born in 1943 **60 years old** n = 655
Height (cm)	179 (7)	166 (7)	178 (7)
Weight (kg)	85.6 (13.3)	70.5 (13.0)	86.3 (12.9)
BMI (kg/m2)	26.6 (3.7)	25.6 (4.5)	27.1 (3.7)
Waist (cm)	94.5 (9.9)	83.1 (11.4)	97.0 (10.0)
Hip (cm)	101.3 (6.6)	99.7 (9.1)	102.0 (6.5)
Waist/hip ratio	0.93 (0.06)	0.83 (0.07)	0.95 (0.06)
BMI ≥ 25 (%)	63.4	45.9	71.6
BMI ≥ 30 (%)	15.3	15.1	16.6
Waist ≥ 80 cm (%)	95.1	56.1	98.2
Waist ≥ 88 cm (%)	76.6	29.8	84.0
Waist ≥ 94 cm (%)	51.3	16.5	60.5
Waist ≥ 102 cm (%)	21.7	7.2	29.8
Waist/hip ratio >0.85 (%)	91.9	37.6	95.9
Waist/hip ratio >0.90 (%)	72.6	15.0	78.8

### Blood pressure and diabetes

Mean blood pressure levels and prevalence of hypertension as well as treatment for hypertension are summarised in Table 4. In general, physician measurement of blood pressure (after breakfast) was higher than the automatic blood pressure measurements (before breakfast by a nurse), except for the diastolic blood pressure in 50-year-old women. Hypertension was observed in one third of the women aged 50 years, almost half of the men aged 50 years and two third of the men aged 60 years. Known diabetes (Table 4) was most common among the 60-year-old men (6.4%) and more common among the 50-year-old men (4.0%) than among the 50-year-old women (2.0%). The prevalence of increased fasting plasma glucose varied between 4 and 33% depending on age, gender, and which cut-off level was employed (Table 4).

**Table 4:** Blood pressure levels (means and standard deviation), the prevalence of hypertension and drug treatment for hypertension.  Prevalence of diabetes and increased fasting plasma glucose.

Variable	**Men** born in 1953 **50 years old** n = 595	**Women** born in 1953 **50 years old** n = 667	**Men** born in 1943 **60 years old** n = 655
SBP, mm Hg, automatic	129.3 (17.8)	123.1 (19.0)	139.8 (21.0)
SBP, mm Hg, physician	134.7 (17.6)	130.7 (18.6)	143.8 (19.6)
DBP, mm Hg, automatic	83.9 (10.9)	82.6 (10.8)	85.1 (11.3)
DBP, mm Hg, physician	84.9 (10.4)	80.8 (10.2)	85.2 (10.5)
Drugs for hypertension (%)	7.1	9.6	22.5
Hypertension (%)	46.2	36.0	66.7
Known diabetes (%)	4.0	2.0	6.4
Fp-glucose ≥ 6.1 mmol/l (%)	10.2	4.2	17.0
Fp-glucose ≥ 5.6 mmol/l (%)	26.1	10.4	32.6

SBP = systolic blood pressure. DBP = diastolic blood pressure phase 5. Hypertension was defined as a physicians diagnosis of hypertension and/or SBP ≥ 140 and/or DBP ≥ 90 (physician measurement), and/or treatment for hypertension.

Fp = fasting, plasma. Fp-glucos ≥ 6.1 and ≥ 5.6 respectively includes those with known diabetes.

To convert p-glucose from mmol/l to mg/dl multiply by 18.

Known diabetes = those who have been told by their doctors that they have diabetes

### Lifestyle habits (smoking, snuff, coffee, alcohol, stress, and physical exercise)

Smoking (Table 2) was more common among the 50-year-old women (26%) than among the 50-year-old men (22%) and the 60-year-old men (15%). Snuff (wet tobacco) was used by 19% of the 50-year-old men and by 17% of the 60-year-old men but by only 3% of the women. Coffee drinking was extremely common: 89–97% of the participants proved to be a daily coffee drinker, with coffee drinking most common among the 60-year-old men. Perceived continuous stress (grade 4 and 5, Table 2) was more common among the women (23%) than among the men (17% among the 50-year-old men and 10% among the 60-year-old men). Regular physical exercise (grade 3–4, see examination procedures) was remarkably similar (24%, in the three cohorts).

### Lipids

Lipid levels and the prevalence of dyslipidaemia using different cut-off levels are listed in Table 5. Mean values of cholesterol were strikingly similar (5.4–5.5 mmol/l) in the three cohorts though women had higher HDL cholesterol levels and lower triglyceride levels than men. Only a minority (3–5%) of the men and women had desirable cholesterol levels, i.e. below 4.0 mmol/l. The prevalence of dyslipidaemia varied greatly depending on the cut-off levels used. Drug treatment for hyperlipidaemia (mainly for hypercholesterolaemia) was uncommon among the 50-year-olds (3–4%) compared with the 60-year-old men (14.5%). Only 2–4% of the participants reported that they were on diet solely because of increased lipid levels.

**Table 5:** Lipids (means and standard deviation) and prevalence of hyperlipidaemia/dyslipidaemia using various cut-off points.  Treatment for hyperlipidaemia.

Variable	**Men** born in 1953 **50 years old** n = 595	**Women** born in 1953 **50 years old** n = 667	**Men** born in 1943 **60 years old** n = 655
S-cholesterol mmol/l	5.50 (1.01)	5.44 (0.93)	5.38 (0.93)
HDL-cholesterol mmol/l	1.45 (0.38)	1.85 (0.45)	1.52 (0.39)
LDL-cholesterol mmol/l	3.31 (0.87)	3.05 (0.88)	3.18 (0.83)
S-triglycerides mmol/l	1.71 (1.18)	1.24 (1.14)	1.54 (0.92)
S-cholesterol <4.0 (%)	4.7	2.9	5.1
S-cholesterol ≥ 5.0 (%)	69.4	69.5	67.1
S-cholesterol ≥ 6.0 (%)	32.4	24.6	26.0
HDL-cholesterol ≤ 1.0 (%)	11.8	1.4	8.0
HDL-cholesterol ≥ 1.6 (%)	34.1	73.4	40.4
LDL-cholesterol ≤ 2.0 (%)	6.9	11.5	8.6
LDL-cholesterol ≥ 4.0 (%)	22.8	14.2	18.7
S-triglycerides ≥ 1.7 (%)	38.2	17.3	22.4
Treatment, drugs (%)	3.9	2.9	14.5
Treatment, diet only (%)	2.2	2.4	4.1

S = serum. HDL = high-density lipoprotein. LDL = low-density lipoprotein.

To convert cholesterol from mmo/l to mg/dl multiply by 38.7.

To convert triglycerides from mmol/l to mg/dl multiply by 88.6

### The metabolic syndrome

**Figure 1:**
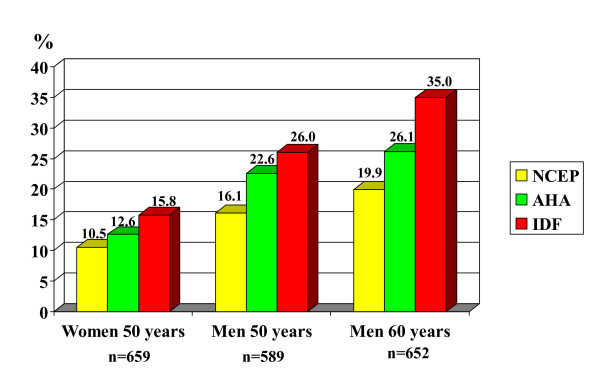
Prevalence (%) of the metabolic syndrome using three definitions.

Three definitions for MetSyn were used for the populations in this study: the NCEP 2001 [12], the AHA 2005 [6] and the IDF 2005 [13] (Table 1). We followed the definitions exactly, i.e. the NCEP definition does not include treatment for hypertension while the other two definitions include that. The WHO definition [11] was not used because we did not measure microalbuminuria and insulin resistance. Blood pressure levels, which were measured automatically, were used for the cut-off levels (≥ 130/85). The prevalence of the MetSyn (Figure 1) increased from 10.5 to 15.8% among the 50-year-old women, from 16.1 to 26% among the 50-year-old men and from 19.9 to 35% among the 60-year-old men depending on which definition was selected. The figures were always lowest with the NCEP definition and highest with the IDF definition.

### Number of risk factors

**Figure 2:**
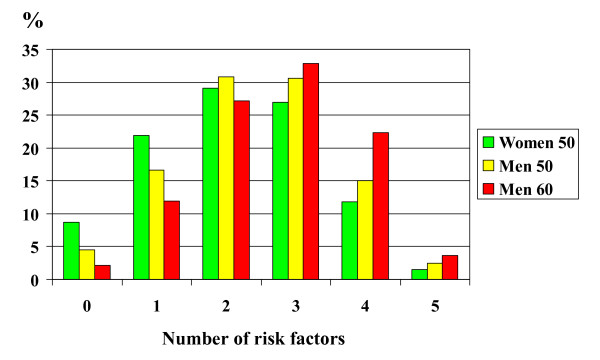
The frequency distribution (%) in relation to number of risk factors (smoking, hypertension, dyslipidaemia, obesity and increased fasting blood glucose/diabetes) in the three cohorts.

The following definitions of five risk factors were used: Smokers were persons who smoked or had quit smoking less than one month before they mailed the questionnaire. Hypertension/high blood pressure was defined as a physician's diagnosis of hypertension and/or systolic blood pressure (physician measurement) ≥ 130 mm Hg and/or diastolic blood pressure ≥ 85 mm Hg, i.e. the levels used for all three definitions of the metabolic syndrome (Table 1). Dyslipidaemia was defined as S-cholesterol ≥ 5.2 mmol/l and/or HDL cholesterol ≤ 1.03 mmol/l for men and ≤ 1.29 mmol/l for women (IDF, Table 1) and/or drug treatment for hyperlipidemia. Obesity was defined as BMI ≥ 30 and/or waist circumference ≥ 94 cm for men and/or ≥ 80 cm for women (IDF, Table 1). Impaired glucose tolerance was defined as known diabetes and/or fasting plasma glucose ≥ 5.6 mmol/l (AHA and IDF, Table 1). When adding these risk factors together, very few of the participants showed zero risk factors (overall only 5%, Figure 2). In the separate cohorts 9% of the 50-year-old women, 5% of the 50-year-old men and 2% of the 60-year-old men had no risk factors. Furthermore, very few showed evidence of all five risk factors: 2% among the 50-year-old women, 3% among the 50-year-old men and 4% among the 60-year-old men. Most of the participants had 2–3 risk factors (56% of the 50-year-old women, 61% of the 50-year-old men and 60% of the 60-year-old men.

## Discussion

The present study investigated the prevalence of cardiovascular risk factors in random samples of middle-aged Swedish men and women examined in the beginning of the 21^st ^century. Using recent definitions of desirable levels, very few (2–9%) of the participants presented no risk factors at all. Moreover, we found that the prevalence of the MetSyn varies substantially depending on which definition is used. More precisely Metsyn varied from 11 to 16% among 50-year-old women, from 16 to 26% among 50-year-old men and from 20 to 35% among 60-year-old men.

There are two limitations that need to be acknowledged and addressed regarding the present study. The first limitation concerns that our study used a cross-sectional design and thus the predictive value of the MetSyn for the development of cardiovascular disease and diabetes cannot be determined because we do not yet have follow-up data. Another limitation is that our populations were not representative of the general population because only two thirds of those invited actually participated. In the first study of 50-year-old men in Gothenburg in 1963 the participation rate was 88% [2]. In later population studies in Gothenburg the participation rate dropped to 76% in 1983 [5] and to 65–69% in the GOT-MONICA project in 1995 [14]. It is known that mortality is higher among non-participants than among participants in the Gothenburg population studies [15,16]. Analyses of non-participants in other population studies are almost non-existent.

Smoking among 50-year-old men has decreased from 56% in 1963 to 22% in the present study. Twenty-two percent is among the lowest rates reported in developed countries [17]. Among the 50-year-old women, 26% were smokers. This percent value could be compared with the final survey of the international MONICA study [17] where smoking rates among women were lowest in Lithuania (5%) and highest in Denmark (42%).

The mean serum cholesterol level of 5.4–5.5 mmol/l in our study ranked in the middle between the lowest (4.5 mmol/l in China) and the highest level (6.3–6.6 mmol/l in Switzerland) in the MONICA study [17]. The higher HDL cholesterol level in our study versus the previous Gothenburg studies [14] may be explained by methodological differences (see Methods) as well as by a higher level of physical activity of the cohorts in our study. In our cohorts 24% had regular or intense physical exercise during leisure time, whereas in the previous Gothenburg cohorts (using the same methodology as in our study) from 1985–1995 [14] the figures were 8–11% for women aged 45–54 years, 13–23% for men aged 45–54 years, and 12–19% for men aged 55–64 years. In comparison with the previous Gothenburg cohorts, the prevalence of obesity, as measured by BMI, has increased slightly among the participants in our study [14].

Using cut points for cardiovascular risk factors as described in the IDF definition of the MetSyn [13], together with smoking and cholesterol ≥ 5.2 mmol/l (≥ 200 mg/dl) very few (2–9%) of the participants had no risk factors at all. In the recent Swedish INTERGENE study [18] 10% of the men and 13% of the women had "optimal" risk factor status. The Norwegian HUNT study [19] reported similar findings. The researchers of the HUNT study concluded that if the 2003 European guidelines on prevention of cardiovascular diseases are implemented, most Norwegians (which they stated to be one of the healthiest populations in the world) would be classified as at high risk for fatal cardiovascular disease [20].

Using different definitions of the MetSyn the prevalence of the MetSyn was found to vary (as expected) in our study as well as in two German studies [21,22] and one Greek study [23], although the figures were higher in the German studies than in our study when comparing similar age cohorts. The German GEMCAS study [22], however, was not a strict population study but participants were those who visited general practitioners. Even Norwegians have a slightly higher prevalence of the MetSyn [20] using the AHA [6] and the IDF [13] definitions than the men and women of similar ages in our study. In Western societies the MetSyn is more common in men than in women, but in a Chinese [24] and an Arab population [25] it was more common in women than in men.

Especially after the introduction of the IDF definition [13] of the MetSyn, there has been an ongoing debate about its usefulness in clinical practice and as a predictor of cardiovascular disease and diabetes [26,27]. It has also been suggested that the syndrome should be dumped entirely [28]. Recently, it was reported that the MetSyn (NCEP criterion) was negligibly linked to incident vascular disease in the elderly [29]. Fasting blood glucose alone was a better predictor of incident diabetes than the MetSyn [29]. In an accompanying editorial [30], the clinical usefulness of the syndrome was questioned. However, another Swedish study [31] concluded that the MetSyn predicts cardiovascular mortality in 50-year-old men, even when taking established risk factors (e.g., smoking and elevated cholesterol) into account.

## Conclusion

This study provides up-to-date information about the prevalence of cardiovascular risk factors and the MetSyn in middle-aged Swedish men and women. Our study reveals that risk factor status has improved, especially regarding smoking. The prevalence of smoking, at least in men, is among the lowest in the world. Based on our findings and those from other studies, the usefulness of the MetSyn is suspect, primarily because it creates uncertainty about which definition to use.

## Competing interests

The authors declare that they have no competing interests.

## Authors' contributions

All authors participated in the design of the study and the examination of the participants. LWe drafted the manuscript and performed the statistical analyses. All authors read and approved the final manuscript.

## Pre-publication history

The pre-publication history for this paper can be accessed here:


